# History of Trauma Exposure and Post-Traumatic Stress Spectrum Symptoms in a Sample of Bariatric Surgery Candidates: Clinical Correlates and Association with Maladaptive Eating Behaviours

**DOI:** 10.3390/ijerph23010106

**Published:** 2026-01-13

**Authors:** Claudia Carmassi, Sara Fantasia, Andrea Bordacchini, Berenice Rimoldi, Debora Andreoli, Gabriele Massimetti, Marly Simoncini, Lorenzo Conti, Valerio Dell’Oste

**Affiliations:** 1 Department of Clinical and Experimental Medicine, University of Pisa, 56127 Pisa, Italy; dr.fantasiasara@gmail.com (S.F.); andreabordacchini@gmail.com (A.B.); bererimo@gmail.com (B.R.); d.andreoli1@studenti.unipi.it (D.A.); gabriele.massimetti@unipi.it (G.M.); marlysimoncini@virgilio.it (M.S.); lorenzo.conti.terp@gmail.com (L.C.); valerio.delloste@gmail.com (V.D.); 2 Department of Mental Health and Addiction, Azienda USL Toscana Nord-Ovest, 55100 Lucca, Italy

**Keywords:** post-traumatic stress symptoms, obesity, bariatric surgery, eating behaviour, emotional eating, grazing

## Abstract

Obesity is a growing health concern in Western countries and the link between obesity and mental disorders has been extensively studied, although less attention has been paid to post-traumatic stress spectrum symptoms (PTSS). This observational study aimed at exploring the correlations between lifetime trauma exposure and its related PTSS and maladaptive eating behaviours in obese patients who are candidates for bariatric surgery. A total of 189 obese candidates for bariatric surgery were recruited at the Azienda Ospedaliero-Universitaria Pisana (AOUP) and assessed by the Trauma and Loss Spectrum—Self-Report (TALS-SR Lifetime Version), Emotional Eating Scale (EES), Night Eating Questionnaire—Italian Version (I-NEQ), Grazing Questionnaire (GQ), and Eating Disorder Examination (EDE-Q), Eating Disorder Inventory (EDI). Based on the TALS-SR (Lifetime Version) scores, patients were dichotomised as either with low PTSS scores or high PTSS scores. Results showed high PTSS scores in more than a third of the sample (36.5%), with these individuals showing statistically significant higher total and EES domain scores, total and controllability GQ domain scores, I-NEQ mood/sleep domain scores, and EDE-Q shape concern domain score. A logistic regression showed an association between higher burden of emotional eating and greater probability of lifetime PTSS. PTSS appear to be frequent among bariatric surgery candidates and are related with maladaptive eating behaviours, suggesting accurate evaluation to optimise surgical outcomes.

## 1. Introduction

Obesity is a chronic, complex disease characterised by an excessive accumulation or abnormal distribution of adipose tissue that can affect an individual’s health [1,2]. Obesity is one of the leading causes of death and disability in Western countries and, particularly in the European Region, contributes to more than 1.2 million deaths per year. Obesity is associated with a significantly increased risk of developing chronic diseases such as cardiovascular disease, type 2 diabetes mellitus, and cancer, and leads to a reduction in life expectancy of 2 to 10 years, depending on the degree of obesity [3]. Obese subjects are also exposed to considerable stigma and the occurrence of psychopathological symptoms [4].

People with a mental disorder have a two to three times higher risk of developing obesity than the general population, and the prevalence of mental illness in people with obesity has been shown to be between 30 and 70% [5]. This co-occurrence may suggest a complex, bidirectional relationship between these conditions, including genetic, environmental, and disease-related factors [4,5,6]. The most studied associations include attention-deficit/hyperactivity disorder (ADHD) [6,7,8], mood disorders [4,5,6,9,10,11,12], schizophrenia [5,13,14], post-traumatic stress disorder (PTSD) [15,16,17], alcohol use disorder [6], and binge eating disorder (BED) [18,19].

Bariatric surgery has previously been recommended among obese patients for individuals with a body mass index (BMI) ≥ 40 kg/m^2^, or ≥35 kg/m^2^ with significant obesity-related comorbidities, who have not achieved sufficient weight loss through structured non-surgical interventions as part of a multidisciplinary program that includes nutritional, psychological, and medical assessment and aims to support long-term behavioural and metabolic change [20,21]. In accordance with the most recent guidelines, bariatric surgery may also be recommended for patients with a body mass index (BMI) between 30 and 39.4 kg/m^2^ who have substantial comorbidities [21]. The benefits of bariatric surgery include not only weight loss and benefits on cardiometabolic profile, but also significant improvements in patients’ mental health. In particular, the reduction in depressive and anxiety symptoms following surgery contributes positively to quality of life [21,22,23]. However, available data indicate that special attention must be paid to the psychopathological framework of patients who are candidates for bariatric surgery, as they have to make long-term behavioural changes following surgery and furthermore some studies show an increased risk of self-harm, suicide, and alcohol abuse after surgery. For this reason, it is essential to consider not only overt diagnoses but also subclinical, often unrecognised psychopathological aspects that can have a significant impact on the occurrence of obesity and negatively influence the outcome of surgery [22,24,25,26].

Notably, problematic eating behaviours such as emotional eating, grazing, night eating, and binge eating can be crucial for the occurrence of obesity and could be signs of psychiatric comorbidities in obese patients [12,27]. Nicholson pointed out as early as 1946 that emotional tension and psychoneurotic states can contribute to the development of obesity [28]. Data from the late 1980s showed that eating is used as a strategy to relieve negative emotions such as anger, depression, loneliness, boredom, and anxiety. However, there was also initial evidence that positive emotions can also trigger eating behaviours [26,29]. Nowadays, emotional eating, also known as comfort eating, is not considered an eating disorder, but rather an eating behaviour that is influenced by habits, stress, emotions, and personal feelings related to food. Emotional eaters tend to choose high-energy, nutrient-poor, and particularly palatable food in response to stressors, leading to an increase in body weight and difficulty losing weight over time [30]. Data suggest that 25.4% to 40% of bariatric surgery candidates have a higher propensity for emotional eating [31].

Grazing is a type of maladaptive eating behaviour characterised by the repeated consumption of rather small/moderate amounts of food. It may be accompanied by a sense of loss of control overeating, which defines the “compulsive” subtype. Varying prevalences have been observed in different studies of bariatric patients, ranging from 17 to 59.8% [32,33]. After bariatric surgery, some authors have suggested patients may consider grazing as a healthy eating behaviour characterised by a conscious choice of foods consumed frequently in small amounts during the day [34]. However, this view changes when considering the compulsive subtype. This subtype appears to occur more frequently in the obese population, whereas grazing in healthy individuals is not associated with a perception of loss of control. In addition, in the bariatric population, up to 80% of binge eaters reported that they experience a loss of control over their eating in the postoperative period, when objectively large binge eating episodes are no longer physically possible, and shift to grazing-like eating behaviour [35,36].

Night eating syndrome (NES) is another important dimension to evaluate when dealing with maladaptive eating behaviours. First described in 1955 and defined by the presence of anorexia in the morning, hyperphagia in the evening, and insomnia [37], it was later integrated with nocturnal ingestions [38]. In its original conceptualisation, NES was considered a peculiar stress response typical of some obese patients and closely related to the overeating that contributed to their obese state [37]. Recently, it was included for the first time in the fifth edition of the *DSM* under “other specified feeding or eating disorders” [37,39]. Although epidemiological studies indicate that there is no direct link between NES and obesity, studies of clinical populations suggest that the prevalence is higher in patients who are overweight or obese [40].

Post-traumatic stress disorder (PTSD) is a psychopathological condition that may occur following the direct or indirect exposure of a traumatic event involving actual or threatened death, serious injury, or sexual violence [39]. It is characterised by a tendency towards a chronic course, a poor response to pharmacological therapy, and a significant impairment of occupational, social, family, and other functions, which leads to a general deterioration in quality of life and an increased risk of suicide [41,42,43,44,45]. According to the literature, people exposed to major traumatic events in line with the *DSM* definition may not fulfil all diagnostic criteria of PTSD but still experience significant functional impairment and the need for support services and specific treatments. The role of so-called low-threshold events (such as divorces, financial problems, illnesses, abortions, sexual harassment, occupational failures, etc.) has also been emphasised in determining post-traumatic stress reactions [46,47,48]. These findings support a multidimensional approach to the psychopathology of PTSD that considers not only the full-blown manifestation of the disorder but also the subclinical spectrum of post-traumatic stress spectrum symptoms (PTSS) that includes atypical manifestations, personality traits, and behavioural manifestations associated with the disorder.

The literature stresses the role of exposure to traumatic events and PTSD in the development of obesity. This link is of particular interest given that recent research shows how PTSD increases the risk of metabolic and cardiovascular disease [15,17,49]. Although this relationship has been studied more in war veterans [50,51] and in individuals exposed to early life trauma [16,52,53,54], it has also been studied in representative samples of the general population, i.e., individuals exposed to different types of trauma [17,55]. The link between PTSD and obesity appears to be mediated by a more complex mechanism, although this is not yet fully understood. PTSD appears to influence body weight through the simultaneous interaction of biological and behavioural processes [56]. On the counterpart, maladaptive eating behaviours have been reported as psychopathological effects of trauma exposure [57].

Despite the scientific evidence clearly demonstrating a link between PTSD and obesity, fewer data have specifically examined the prevalence of lifetime trauma and PTSS in bariatric surgery candidates. Currently available data show high rates of childhood trauma, particularly maltreatment, in the bariatric population [58,59] and a prevalence of PTSD between 3.1 and 11% [60,61]. However, several authors have pointed out that these rates may be an underestimate, as patients may not report exposure to child maltreatment or underestimate symptoms related to traumatic life events [59]. To our knowledge, the data on the effects that trauma exposure or the development of PTSD may have on the outcome of surgery are even more limited. Ikossi et al. [62] identified 24 veterans who underwent gastric bypass and were diagnosed with PTSD. They showed weight loss comparable to those who did not suffer from PTSD and a fluctuation in PTSD symptoms over time that did not allow for defining the impact of the surgery on psychiatric symptoms. In contrast, Porter et al. [63] found that subjects with a history of trauma were less likely to adhere to the behavioural recommendations required to maintain postoperative weight loss, such as support visits, nutritional visits, and exercise programmes.

The primary aim of this study was to evaluate the prevalence of post-traumatic stress symptoms in a sample in this population. The secondary outcome of our study was to identify possible correlations between post-traumatic stress symptoms and maladaptive eating behaviours reported by the study population and to investigate which specific maladaptive eating behaviour was more strongly related to a higher PTSS burden.

## 2. Materials and Methods

### 2.1. Study Sample

The present study is an observational study of 189 subjects who were candidates for bariatric surgery (Roux-en-Y gastric bypass or sleeve gastrectomy) at the Multidisciplinary Centre for the Diagnosis and Treatment of Obesity of the Azienda Ospedaliera Universitaria Pisana (AOUP) and who were referred to the Psychiatric Clinic of the AOUP as part of the preoperative assessment. In accordance with national and international guidelines, the preoperative multidisciplinary assessment of bariatric surgery candidates also includes a mental health assessment to identify any psychiatric disorders or maladaptive psychosocial factors that could influence the preoperative and postoperative outcome or even contraindicate surgery.

Patients were consecutively recruited from July 2024 up to June 2025, in the framework of a multidisciplinary collaborative group for the treatment of obese patients running between clinicians of the Psychiatric, Internal Medicine, and Bariatric Surgery Units of the AOUP since 2018. Exclusion criteria included being under 18 or over 75 years of age, lack of Italian language skills or other limitations in verbal communication that prevented the subject from following the study protocol, mental disability, any current or lifetime mental disorders assessed by *DSM-5-TR* criteria by psychiatrist or psychiatry residents, and/or undergoing psychopharmacological treatment. All participants were informed about this study and had the opportunity to ask questions before giving written informed consent. Every patient who met the inclusion criteria accepted voluntary enrolment in this study. This study was conducted in accordance with the Declaration of Helsinki and approved by the AOUP Ethics Committee (protocol code: BARPSIC, ID 27023, approved on 30 July 2024).

Sociodemographic and clinical information was collected in a data sheet that included the following information: gender, age, weight, height, BMI, degree of obesity, marital status, education level, employment status, medical comorbidities, psychiatric diagnoses, and current psychopharmacological treatment.

Psychiatric assessment was performed by psychiatrists or psychiatry residents and included the following self-administered questionnaire: TALS-SR Lifetime Version (Trauma and Loss Spectrum—Self-Report) [48], EES (Emotional Eating Scale) [29], I-NEQ (Italian Night Eating Questionnaire) [40,64], GQ (Grazing Questionnaire) [36], EDE-Q (Eating Disorder Examination-Questionnaire) [65,66], and EDI (Eating Disorder Inventory) [67,68,69].

### 2.2. Instruments and Assessments

The TALS-SR (Lifetime Version) [48] is a self-administered questionnaire that takes a multidimensional approach to the post-traumatic stress spectrum by considering its three dimensions: potentially traumatic events, acute peritraumatic reactions, symptoms of the post-traumatic stress spectrum (PTSS), behavioural manifestations, and temperamental and personality factors. The TALS-SR (Lifetime Version) has proven to have good psychometric properties in terms of validity and reliability. The questionnaire consists of 116 dichotomously answered items divided into 9 domains: loss events, grief reactions, potentially traumatic events, reaction to losses or upsetting events, re-experiencing, avoidance and numbing, maladaptive behaviour, arousal, and personal characteristics/risk factors. In line with previous studies [70,71,72,73,74], the use of specific items from the TALS-SR (Lifetime Version), carefully selected to match the diagnostic criteria for PTSD according to the *DSM-5-TR*, allows for the assessment of the presence of full or partial PTSD, ensuring a complete understanding of the spectrum of post-traumatic stress reactions regarding the domains related to potential traumatic events listed in the TALS-SR (Lifetime Version).

The EES [29] is an instrument that allows for a detailed assessment of the relationship between unpleasant emotional states and overeating. The questionnaire includes 25 unpleasant emotions and respondents are asked to indicate for each emotion the degree to which it stimulates the desire to eat. The answers are given on a Likert scale (no desire to eat, slight desire to eat, moderate desire to eat, strong urge to eat, overwhelming urge to eat). In addition to the mean value, the values for three subscales are also assessed: anger/frustration, anxiety, and depression. The questionnaire showed internal consistency and stability over time.

The I-NEQ [40,64] is the most widely used screening instrument for assessing NES. It consists of 14 items arranged on a Likert scale with scores ranging from 0 to 4 and examines four main dimensions: morning anorexia, evening hyperphagia, mood/sleep, and nocturnal ingestion. The I-NEQ is an instrument with acceptable psychometric properties and good test–retest reliability. In addition, there is considerable psychometric similarity with the original version of the NEQ and its subsequent validations.

The GQ [36] is a questionnaire that was developed to reflect the unplanned, continuous, and repeated intake of lesser amounts of food over extended periods of time. In addition, specific items can be used to assess whether this behaviour is associated with the perception of a loss of control overeating. The questionnaire is designed to distinguish grazing behaviour from both binges and planned snacks. It consists of 8 items rated on a 5-point Likert scale (from 0 = never to 4 = all of the time): 5 assess grazing behaviour and 3 assess the perception of loss of control overeating. In this way, two subscales are defined, grazing behaviours and controllability, both with good internal consistency. The GQ has indeed shown good psychometric properties in terms of reliability and validity.

The EDE-Q [65,66] is the self-administered version of the Eating Disorder Examination (EDE) interview, which is considered the gold standard for the assessment of eating psychopathology. The EDE-Q uses 28 Likert-structured items to provide a total score based on four main subscales: restraint, eating concern, shape concern, and weight concern. These subscales are constructed to reflect the severity of the key features of eating disorder psychopathology. The questionnaire showed good psychometric properties: internal consistency, stability over time, test–retest reliability, convergent validity, and sensitivity to change.

The EDI [67] is a self-administered questionnaire that provides a multidimensional approach to the psychopathology of eating disorders by assessing attitudinal and behavioural aspects. It consists of 64 items with answers structured on a Likert scale (often, usually, rarely, never) divided into 8 subscales: drive for thinness, bulimia, body dissatisfaction, ineffectiveness, perfectionism, interpersonal distrust, interoceptive awareness, and maturity fear. The EDI has proven to be a valid and reliable test with good internal consistency.

### 2.3. Statistical Analysis

All variables—demographic, clinical, and assessment test results—were primarily analysed using descriptive statistics. Continuous variables were reported as mean ± standard deviation (SD), while categorical variables were expressed as percentages.

Based on the TALS-SR (Lifetime Version) results, we differentiated the sample into “Low PTSS score” if they reported lifetime endorsement of none or less than two symptom domains criteria for the diagnosis of PTSD and “High PTSS score” if they reported lifetime endorsement of at least 2 symptom domains of PTSD as described by criteria B, C, D, and E of *DSM-5-TR* and correspondently evaluated by the means of TALS (lifetime exposure) domains IV, V, VI, VII, and VIII. We then compared the analysed variables with those of the Low PTSS score group. Comparisons of sociodemographic categorical variables (gender, marital status, educational level, employment status) and clinical categorical variables (degree of obesity, medical comorbidities, psychiatric diagnoses, current psychopharmacological treatment) between the Low PTSS score and High PTSS score groups were performed using chi-square. In addition, the independent Student *t*-test was used to compare continuous variables (age, weight, height, BMI) between the same groups.

Comparisons between the Low PTSS score and High PTSS score groups regarding sociodemographic and clinical variables and the results of the questionnaires on eating behaviour were carried out with the Mann–Whitney U-test, followed by Bonferroni correction. Furthermore, a multiple logistic regression model was employed to determine which variables were more strongly linked to the risk of being included in the High PTSS score group among those for which the univariate comparisons in mean scores were statistically significant. All tests were two-sided tests and a *p*-value ≤ 0.050 was considered statistically significant. All statistical analyses were performed with the Statistical Package for the Social Sciences, Version 26.0 (SPSS Inc., New York, NY, USA).

## 3. Results

The total sample included 189 subjects, 66% of whom were females. The average age of the sample was 47.12 years ± 10.46, while the average weight was 122.98 kg ± 22.45. Notably, 71.3% of the participants suffered from grade III obesity. Most of the sample was married or cohabiting (74.5%), had a secondary school diploma (46.6%), and was employed (70.4%). Medical comorbidity was reported by 44.1% of subjects. Statistically significant differences regarding demographic data emerged on weight and employment status between those with a low PTSS score and high PTSS, with the latter group showing significantly lower employment levels (*p* = 0.039) and lower weight (*p* = 0.018) compared to the Low PTSS score group. The demographic and clinical characteristics of the total sample are shown in Table 1.

Regarding lifetime traumatic exposure: at least one lifetime potentially traumatic event was reported by 97.8% (N = 185) of the sample. The most commonly reported events in the total sample were in the category of loss events (such as death of a close friend or family member, pregnancy loss or death in childbirth, and others), with the death of a close friend or a family member as the most common one (84.6%); in terms of interpersonal potentially traumatic events, the most represented was family conflicts (23.28%), followed by bullying (22.75%) and sexual trauma (undesired advances—16.9%; sexual assault—9.5%; rape—3.2%), other relevant traumas represented in our sample were serious illness (20.1%), physical assault (15.9%), severe accident (15.9%), mass trauma (13.2%), being arrested or charged (4.8%), and war exposure (4.2%). Analysis of the TALS-SR (Lifetime Version) results showed that 7.4% (N = 14) of participants reported symptoms consistent with a lifetime diagnosis of PTSD and 29.1% (N = 55) with lifetime partial PTSD, adding up to a percentage of 36.5% (N = 69) of subjects included in the High PTSS score group. The mean scores of the TALS-SR (Lifetime Version) domains and the endorsement of the *DSM-5-TR* criteria for PTSD are shown in Table 2.

**Figure 1:**
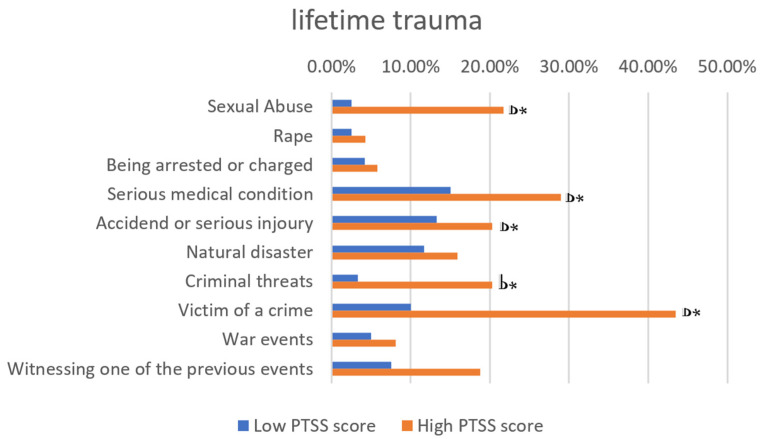
Types of trauma to which the study participants reported exposure. * *p* ≤ 0.050.

Dividing the sample into Low PTSS score (N = 120, 63.5%) and High PTSS score (N = 69, 36.5%) groups, both the groups reported loss events as the most frequent potentially traumatic. However, the High PTSS score group was significantly more likely to report a higher lifetime exposure of some major traumatic events: sexual abuse (21.7% vs. 2.5% *p* < 0.001), serious medical condition (29.0% vs. 15.0%, *p* = 0.021), criminal threats (43.50% vs. 10.0%, *p* < 0.001), being victim of a criminal act (43.5% vs. 10.0%, *p* < 0.001), being witness of a potentially traumatic event (18.8% vs. 7.5%, *p* = 0.019) (Figure 1).

**Figure 2:**
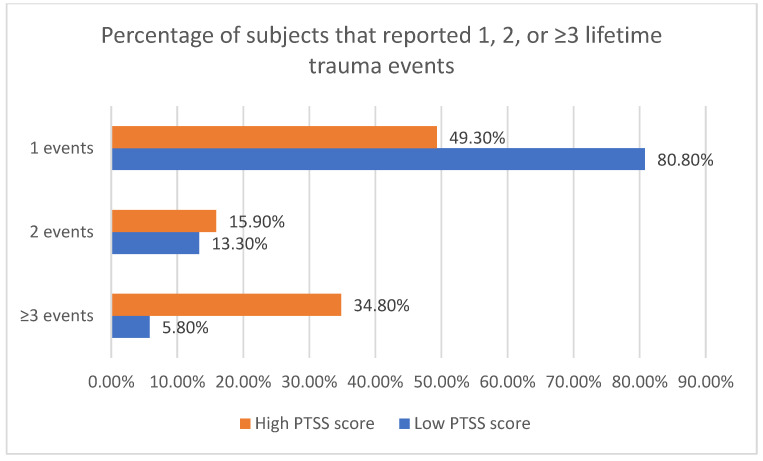
Percentage of subjects that reported 1, 2, or ≥3 trauma events in the two groups.

Individuals in the High PTSS score group were more likely to report a greater lifetime exposure to trauma compared to those with a lower PTSS score. Specifically, 34.8% of individuals in the High PTSS score group reported experiencing three or more events (Figure 2).

Patients with a high PTSS score had significantly higher mean scores for the total score and the subscales of the EES, for the *mood/sleep* subscale of the NEQ, for the total score and the controllability subscale of the GQ, and for the *shape* concern subscale of the EDE-Q. No significant differences between the two groups were recorded in the EDI scale. The results of the eating behaviour rating scales for the total sample and the two groups and the significance values of the comparisons between them are shown in Table 3.

Given the significant differences in EES total score means and GQ scale total score between the two groups, a logistic regression analysis was performed to establish whether these two variables were predictive of higher PTSS scores. Within the model, only the EES total score showed significant predictive value. See Table 4 for details.

## 4. Discussion

The results of the present study explored lifetime trauma exposure and its related post-traumatic stress spectrum symptomology among bariatric surgery candidates consecutively recruited without any current psychiatric diagnosis or psychopharmacological treatment. The correlations with maladaptive eating behaviours were explored. Our results show a lifetime trauma exposure reported by more than 90% of the total sample. These data are consistent with the scientific literature, which indicates high rates of exposure to traumatic events in people affected by obesity [15,16,17,49,50,51,52,53,54,55,56]. Based on our analysis, a high PTSS score was found in 36.5% of subjects. This group of subjects includes participants who reported lifetime endorsement of at least two symptomatologic domains of PTSD as described by criteria B, C, D, and E of *DSM-5-TR* and evaluated by the means of TALS (lifetime exposure) domains IV, V, VI, VII, and VIII. This spectrum approach to post-traumatic stress disorder is consistent with our previews work and allows for us to capture subclinical or subthreshold lifetime manifestations of post-traumatic stress disorder, even in non-clinical populations, that could contribute to the eventual development of psychopathology or maladaptive behaviours [72,73,74].

The study population was mainly composed of individuals affected by morbid obesity (grade III) (71.3%), with average body weight and BMI of 122.98 kg and 43.76 kg/m^2^, respectively. Although in the general population obesity rates are similar in both sexes, the study sample shows a female predominance (66.1%) and this, as corroborated by other previous studies [75,76], finds possible explanations in the fact that there is greater social pressure on females to achieve aesthetic standards of thinness and a greater propensity to go to the doctor for weight control [75,76]. The two groups were similar in sociodemographic characteristics, except that unemployment was significantly more common in the PTSS group. This data is in line with the fact that trauma symptomatology is generally associated with the reduced ability to carry out daily activities with consequent significant impairment of work activity [77,78]. Although the literature highlights an increased risk of cardiovascular and metabolic diseases in people with PTSD [15,79,80,81], our study shows no differences between the Low PTSS score group and the High PTSS score group in terms of medical comorbidities. This could be explained by the fact that the main driver of medical comorbidity in our case could be, in fact, obesity.

The number of traumatic events to which a person is exposed has been reported as a risk factor for the development of PTSD [39,77,82,83,84], with interpersonal traumas being strongly correlated with the worst outcomes [85,86]. We observed that those in the Low PTSS score group were more likely to have been exposed to only one potentially traumatic event (80.8% vs. 49.3%, *p* < 0.001), while those in the High PTSS score group were more likely to report three or more potentially traumatic events (43.8% vs. 5.8%, *p* < 0.001). In this context, we cannot estimate the likelihood of the presence of complex PTSD in some subject of our sample, particularly in the subgroup with a high PTSS score, as no data on prolonged or repeated exposure to trauma was collected, representing a possible limitation of the present study.

Regarding the relationship of higher trauma related symptomatology and maladaptive eating behaviour, patients with high PTSS scores showed significantly more emotional eating than the group with low PTSS scores, as detected by EES. This data is in line with the literature, as different studies have shown a stronger tendency to change eating behaviours in response to emotions or stress in the presence of PTSD [87,88]. Indeed, PTSD is strongly correlated with emotional dysregulation [89,90], a transdiagnostic psychopathological dimension characterised by an inability to inhibit inappropriate emotional reactions to internal or external stimuli [91]. Difficulties in emotional regulation may contribute to maladaptive eating behaviours, particularly binge eating disorder and emotional eating [92,93,94]. We believe that the higher burden of emotional eating (as measured by total and subscale scores of the Eating Disorders Examination—Self-Report) in the group of patients characterised by higher PTSS scores could be a reflection of the frequently observed bidirectional relationship between emotional dysregulation and trauma symptoms [39,77,91,95].

Our data show that subjects with high PTSS scores have a significantly higher incidence of grazing behaviour, particularly the compulsive subtype. The compulsive grazing variant has been associated with a higher degree of psychopathology, more pronounced psychological deterioration, and a lower quality of life [96,97]; additionally, although it does not appear to significantly influence BMI, it also plays an important role in influencing the outcome of bariatric surgery, interfering with weight loss and maintenance [32,33,35,98,99,100]. Difficulties in emotional regulation could also be linked to the higher frequency of grazing in the High PTSS score group, since grazing behaviour is frequently carried on as a maladaptive emotional regulation behaviour [101,102] with both perceived mood-enhancing activity and potentially addictive risk due to the effect on the reward system [103,104]. In this context, it is noteworthy that the non-compulsive subtype—specifically the controllability subscale of grazing behaviour, which is explored through questions analysing the inability to stop the intake of food [101] with a sense of loss of control similar to a binge eating episode—exhibits a statistically higher score in the High PTSS score group. This could be linked to an impairment in top-down prefrontal regulatory functions that could be present in post-traumatic stress symptomatology [105].

Distinct considerations could be made regarding the varied outcomes observed on the mood/sleep subscale of the Night Eating Questionnaire (NEQ). Patients with higher PTSS scores exhibited higher scores on the domain typically associated with sleep disturbances, anxiety, and a sensation of sadness during nighttime, as well as the perceived necessity to eat to cope with difficulties in falling asleep. The same result was achieved by Dorflinger et al. [106], who investigated the presence of NES in subjects with PTSD, which as is known have a significantly greater probability of testing positive for sleep disorders [107]. In this context, hyperarousal and autonomic and metabolic dysregulation associated with trauma symptoms may contribute to the establishment of mood and sleep disturbances at night and facilitate night eating as a coping strategy [108]. However, the current analysis cannot establish a definitive causal correlation. Another statistically significant difference in mean scores between the two groups emerged on the shape concern subscale of the Eating Disorder Examination Questionnaire (EDE-Q). This phenomenon may be attributed to the frequent correlation between a history of trauma and difficulties and preoccupations related to body image [109].

Given the statistically significant difference in the total EES score and total GQ score, we performed a logistic regression analysis to establish whether grazing or emotional eating were predictive of higher PTSS at our single timepoint evaluation. In our analysis, a positive and significant predictive value emerged for the EES total score, but not for the GQ total score, indicating that emotional eating and presumably the underlying emotional dysregulation were strongly associated with PTSS [90,110].

Overall, these data in conjunction with the results of the logistic regression seem to indicate that subjects with PTSS are frequently subjected to emotional eating: more specifically, a higher burden of emotional eating is linked with a greater probability of reported lifetime PTSS, with an increased risk of 3% to be included in the High PTSS group for each single-point increase in the EES total score. Interesting results emerged from the differences in BMI and body weight between the two groups, since subjects in the High PTSS score group showed a higher propensity for emotional eating but had a significantly lower average weight compared with subjects with low PTSS scores. In this regard, it is important to note that the High PTSS score group comprises a higher proportion of female subjects; this could be responsible for the statistically significant difference in weight, but not in BMI. Regarding the higher propensity for emotional eating and its link to obesity, it is important to note that such maladaptive eating behaviour is not solely linked to higher BMI but could also simply be a more prolonged history of obesity that, according to updated guidelines, indicates a high burden of disease and justifies a surgical approach to obesity [21].

This finding would suggest the significant role of emotional dysregulation in the link between PTSS and maladaptive eating behaviour that affects body weight [111,112,113]. Recently, it has been proposed as a transnosographic entity in the major mental disorders, following evidence of its essential, though often overlooked, role in several psychopathological conditions [91]. There is substantial overlap between PTSD and emotional dysregulation [91,114,115,116] and it has not yet been possible to definitively clarify whether emotional dysregulation is an entity that precedes trauma, a predictor of symptom severity and maintenance; whether it is a consequence of trauma; or whether trauma and emotional dysregulation have a bidirectional relationship [91]. According to the literature, the impairment of cognitive emotional regulation strategies is crucial for the progression to obesity [117,118], as these subjects would seem more susceptible to adopt compulsive or addictive eating behaviours [119] that may lead to poorer outcomes after surgery [120].

Although further studies focusing on emotional dysregulation in the relationship between PTSS and maladaptive eating behaviour are needed, the scientific literature mentioned above seems to support this hypothesis. Another aspect that emerges from these findings is the possible underestimation in terms of diagnosis and severity of the disorders according to other studies whose methodology are comparable with that of the present study, particularly in terms of recruitment and diagnostic method, although they are at the lower limit of the prevalence range reported in the general literature on bariatric patients [27,121,122,123,124]. An explanation of this phenomenon could be the tendency of patients to downplay their symptoms or omit psychopathological symptoms, which may be because our psychiatric visit in our case is part of the preoperative pathway and may be a barrier to surgery.

Although the present study was not designed to follow the outcome after surgery, it can be hypothesised that patients who report presence of lifetime PTSS may be at risk for poorer weight loss maintenance following bariatric surgery, as suggested by the relationship that our study found between PTSS and preoperative maladaptive eating behaviours that have previously identified as a predictor of weight regain after surgery [32,33,35,98,125,126].

When discussing our results, we need to consider some limitations that could influence the interpretation of the data. First, as suggested by the literature, it is possible that also in our study, the setting of the psychiatric assessment performed as part of the bariatric surgery protocol may have led to underestimation of psychiatric diagnoses and psychopathological elements. Secondly, the assessment was conducted after exposure to the potentially traumatic event, and we have no information about the participants’ condition prior to exposure, limiting our ability to establish causality between the elements that resulted from the analysis; moreover, the cross-sectional design of our study prevents us from inferring any temporal or clear causal correlation between PTSS and maladaptive eating behaviour or post-surgical outcomes. Thirdly, several patients failed to complete the assessment because of the numerous questions and the total length of the tests administered, which resulted in missing data. Furthermore, we used self-report instruments made for the assessment of trauma exposure, potentially leading to an overestimation of the trauma in terms of self-perceived traumatisation [127]. We must also acknowledge the limitations of retrospective trauma reporting [128], which could include both negative and positive recall biases [129] or under-reporting of some types of events [130]. Several known shortcomings of self-report scales on maladaptive eating behaviour should also be kept in mind [131]. Additionally, in interpreting the results of this study, it is important to acknowledge that our sample exclusively comprised candidates for bariatric surgery willing to disclose trauma and undergo psychiatric evaluation. Consequently, this sample may not accurately represent the entirety of bariatric surgery candidates nor of the obese population. Finally, although there is evidence for the role of emotion dysregulation in bariatric patients and PTSD, we did not use questionnaires that specifically assess emotion dysregulation at baseline.

## 5. Conclusions

This study shows a high prevalence of lifetime trauma and of PTSS in bariatric surgery candidates, especially compared to the general population, and suggests an association with problematic eating behaviour. Although further studies are needed to better understand the relationship between PTSS, problematic eating behaviours, and bariatric surgery outcomes, this study highlights the need for careful management and assessment of psychopathological symptoms in bariatric surgery candidates, even in the absence of formal psychiatric diagnoses, with personalised prevention and follow-up interventions to better understand each patient’s maladaptive eating behaviours and optimise the outcomes of surgery.

## Figures and Tables

**Table 1:** Sociodemographic and clinical characteristics of the total sample and comparison between Low PTSS score and High PTSS score groups.

	Total Sample (N = 189)	Low PTSS Score (N = 120)	High PTSS Score (N = 69)	OR	*p*
	Mean ± SD	Mean ± SD	Mean ± SD		
Age (years)	47.12 ± 10.46	46.43 ± 10.00	48.32 ± 11.18		N.s.
Weight (kg)	122.98 ± 22.45	125.46 ± 23.37	118.67 ± 20.21		0.018
Height (cm)	167.44 ± 9.51	168.32 ± 9.07	165.90 ± 10.12		N.s.
BMI (kg/m^2^)	43.76 ± 6.28	44.14 ± 6.63	43.08 ± 5.57		N.s.
	N (%)	N (%)	N (%)		
Degree of Obesity					N.s.
I (BMI = 30–34.9)	6 (3.2%)	3 (2.5%)	3 (4.4%)		
II (BMI = 35–39.9)	48 (25.5%)	28 (23.3%)	20 (29.4%)		
III (BMI ≥ 40)	134 (71.3%)	89 (74.2%)	45 (66.2%)		
Gender				1.42	N.s.
Male	64 (33.9%)	44 (36.7%)	20 (29.0%)		
Female	125 (66.1%)	76 (63.3%)	49 (71.0%)		
Marital Status				0.78	N.s.
Single	22 (11.7%)	15 (12.6%)	7 (10.1%)		
Married/Cohabiting	140 (74.5%)	86 (72.3%)	54 (78.3%)		
Separated/Divorced	21 (11.2%)	15 (12.6%)	6 (8.7%)		
Widowed	5 (2.7%)	3 (2.5%)	2 (2.9%)		
Education Level				1.41	N.s.
Graduate/Post-Graduate	18 (9.6%)	12 (10%)	6 (8.6%)		
High School Diploma	73 (38.6%)	42 (35.0%)	31 (44.9%)		
Lower Secondary School Diploma	88 (46.6%)	63 (52.5%)	25 (36.2%)		
Primary School Certificate/Incomplete Primary School	10 (5.2%)	3 (2.5%)	7 (10.1%)		
Employment Status				1.47	0.039
Employed	133 (70.4%)	88 (73.3%)	45 (65.2%)		
Unemployed	56 (29.6%)	32 (26.7%)	24 (34.8%)		
Medical Comorbidities					N.s.
No	105 (55.9%)	65 (54.2%)	40 (58.8%)		
Yes	83 (44.1%)	55 (61.9%)	28 (41.2%)		

**Table 2:** Mean TALS-SR (Lifetime Version) domains scores and endorsement of *DSM-5-TR* PTSD criteria in the total sample.

	Total Sample (N = 189)
	Mean ± SD
TALS-SR (Lifetime Version)	
I—Loss events	4.02 ± 1.97
II—Grief reactions	7.75 ± 4.85
III—Potentially traumatic events	2.93 ± 2.80
IV—Reactions to losses or upsetting events	4.53 ± 3.70
V—Re-experiencing	1.66 ± 1.78
VI—Avoidance and numbing	1.85 ± 2.22
VII—Maladaptive behaviours	0.34 ± 0.80
VIII—Arousal	0.63 ± 1.18
Tot PTSS score (domains IV + V + VI + VII + VIII)	9.0 ± 7.75
	N (%)
DSM-5-TR diagnostic criteria	
Criterion B—Intrusive symptoms	100 (52.9%)
Criterion C—Avoidance	66 (34.9%)
Criterion D—Negative alterations in cognition and mood	54 (28.6%)
Criterion E—Arousal	37 (19.6%)
PTSS score	
Low PTSS score	120 (63.5%)
High PTSS score	69 (36.5%)

**Table 3:** Scores of the eating behaviour scales in the total sample, with a comparison between the *Low PTSS score* group and the *High PTSS score* group. *p* * = *p*-value adjusted for multiple comparisons using Bonferroni correction.

	Total Sample (N = 189)	Low PTSS Score (N = 120)	High PTSS Score (N = 69)	*p* *
	Mean ± SD	Mean ± SD	Mean ± SD	
EES				
Total Score	19.1 ± 19.55	15.57 ± 17.66	25.04 ± 21.23	0.008
Anger	8.18 ± 9.02	6.50 ± 8.00	11.00 ± 9.97	0.004
Anxiety	6.75 ± 7.04	5.62 ± 6.48	8.63 ± 7.59	0.024
Depression	4.18 ± 4.16	3.44 ± 3.79	5.41 ± 4.48	0.008
NEQ				
Total Score	14.33 ± 5.39	13.57 ± 5.00	15.58 ± 5.8	n.s.
Morning Anorexia	3.30 ± 1.71	3.43 ± 1.60	3.08 ± 1.88	n.s.
Evening Hyperphagia	2.61 ± 2.66	2.51 ± 2.55	3.76 ± 2.87	n.s.
Mood/Sleep	3.08 ± 2.56	2.46 ± 2.40	4.12 ± 2.51	0.01
Nocturnal Ingestions	5.33 ± 2.44	5.16 ± 2.06	5.61 ± 3.00	n.s.
GQ				
Total Score	7.38 ± 7.38	6.37 ± 5.28	8.93 ± 5.01	0.015
Grazing Behaviours	4.91 ± 3.28	4.49 ± 3.33	5.56 ± 3.14	n.s.
Controllability	2.47 ± 2.60	1.88 ± 2.44	3.35 ± 2.44	0.003
EDE-Q				
Global EDE-Q Score	2.28 ± 1.03	2.16 ± 0.97	2.56 ± 0.99	n.s.
Restraint	1.64 ± 1.36	1.56 ± 1.30	1.79 ± 1.47	n.s.
Eating Concern	1.05 ± 1.19	0.91 ± 1.18	1.29 ± 1.20	n.s.
Shape Concern	3.53 ± 1.49	3.29 ± 1.50	3.92 ± 1.38	0.04
Weight Concern	2.98 ± 1.25	2.80 ± 1.25	3.25 ± 1.25	n.s.
EDI				
Total Score	47.17 ± 24.25	43.45 ± 22.70	53.14 ± 25.61	n.s.
Drive for Thinness	7.08 ± 5.96	6.67 ± 6.33	7.75 ± 5.26	n.s.
Bulimia	2.20 ± 2.97	1.97 ± 2.79	2.59 ± 3.25	n.s.
Body Dissatisfaction	15.35 ± 6.80	14.63 ± 6.71	16.53 ± 6.68	n.s.
Ineffectiveness	3.61 ± 4.59	3.20 ± 4.18	4.31 ± 5.17	n.s.
Perfectionism	3.59 ± 3.45	4.22 ± 3.51	4.21 ± 3.51	n.s.
Interpersonal Distrust	4.58 ± 3.90	4.01 ± 3.76	5.50 ± 4.00	n.s.
Interoceptive Awareness	3.65 ± 4.65	3.23 ± 4.44	4.34 ± 4.93	n.s.
Maturity Fears	7.44 ± 4.25	7.30 ± 4.39	7.69 ± 4.02	n.s.

Abbreviations: PTSS = post-traumatic stress symptoms; EES = Emotional Eating Scale; NEQ = Night Eating Questionnaire; GQ = Grazing Questionnaire; EDE-Q = Eating Disorder Examination Questionnaire; EDI = Eating Disorder Inventory. *p* *: all *p*-values are reported with Bonferroni correction and are considered significant if ≤0.050.

**Table 4:** Logistic regression analysis EES total scores associating high PTSS score in the total sample.

Total Score	B (SE)	p *	OR	95% Confidence Interval
Total EES	0.032 (0.011)	0.006	1.032	1.009–1.055
Total GQ	0.062 (0.048)	0.200	1.064	0.968–1.170

Cox & Snell R^2^ = 0.129; Nagelkerke R^2^ = 0.173. Abbreviations: EES = Emotional Eating Scale; GQ = Grazing Questionnaire; OR = odds ratio, *p* *: all *p*-values are reported with Bonferroni correction and are considered significant if ≤0.050.

## Data Availability

The original contributions presented in this study are included in the article. Further inquiries can be directed to the corresponding author.

## Abbreviations

The following abbreviations are used in this manuscript:

ADHD	Attention Deficit Hyperactivity Disorder
AOUP	Azienda Ospedaliero-Universitaria Pisana
BED	Binge Eating Disorder
BMI	Body Mass Index
*DSM-5-TR*	*Diagnostic and Statistical Manual of Mental Disorders, Fifth Edition, Text Revision*
EDE-Q	Eating Disorder Examination-Questionnaire
EDI	Eating Disorder Inventory
EES	Emotional Eating Scale
GQ	Grazing Questionnaire
I-NEQ	Italian Night Eating Questionnaire
IFSO	International Federation for the Surgery of Obesity and Metabolic Disorders
NES	Night Eating Syndrome
NIH	National Institutes of Health
PTSD	Post-Traumatic Stress Disorder
PTSS	Post-Traumatic Stress Spectrum Symptoms
SCID-5	Structured Clinical Interview for DSM-5
SD	Standard Deviation
SPSS	Statistical Package for the Social Sciences
TALS-SR (Lifetime Version)	Trauma and Loss Spectrum—Self-Report
